# A literature review of microRNA and gene signaling pathways involved in the apoptosis pathway of lung cancer

**DOI:** 10.1186/s12931-023-02366-w

**Published:** 2023-02-17

**Authors:** Hanie Abolfathi, Mohadeseh Arabi, Mojgan Sheikhpour

**Affiliations:** 1 Department of Molecular Medicine, Faculty of Medicine, Laval University, Quebec, Canada; 2 Department of Mycobacteriology and Pulmonary Research, Pasteur Institute of Iran, Tehran, Iran; 3 Microbiology Research Center, Pasteur Institute of Iran, Tehran, Iran

**Keywords:** Lung cancer, Gene, miRNA, Signaling pathway, Apoptosis

## Abstract

**Background:**

Lung cancer is one of the leading causes of death in the world and the deadliest of all cancers. Apoptosis is a key pathway in regulating the cell growth rate, proliferation, and occurrence of lung cancer. This process is controlled by many molecules, such as microRNAs and their target genes. Therefore, finding new medical approaches such as exploring diagnostic and prognostic biomarkers involved in apoptosis is needed for this disease. In the present study, we aimed to identify key microRNAs and their target genes that could be used in the prognosis and diagnosis of lung cancer.

**Methods:**

Signaling pathways, genes, and microRNAs involved in the apoptotic pathway were identified by bioinformatics analysis and recent clinical studies. Bioinformatics analysis was performed on databases including NCBI, TargetScan, UALCAN, UCSC, KEGG, miRPathDB, and Enrichr, and clinical studies were extracted from PubMed, web of science, and SCOPUS databases.

**Results:**

NF-κB, PI3K/AKT, and MAPK pathways play critical roles in the regulation of apoptosis. MiR-146b, 146a, 21, 23a, 135a, 30a, 202, and 181 were identified as the involved microRNAs in the apoptosis signaling pathway, and IRAK1, TRAF6, Bcl-2, PTEN, Akt, PIK3, KRAS, and MAPK1 were classified as the target genes of the mentioned microRNAs respectively. The essential roles of these signaling pathways and miRNAs/target genes were approved through both databases and clinical studies. Moreover, surviving, living, BRUCE, and XIAP was the main inhibitor of apoptosis which act by regulating the apoptosis-involved genes and miRNAs.

**Conclusion:**

Identifying the abnormal expression and regulation of miRNAs and signaling pathways in apoptosis of lung cancer can represent a novel class of biomarkers that can facilitate the early diagnosis, personalized treatment, and prediction of drug response for lung cancer patients. Therefore, studying the mechanisms of apoptosis including signaling pathways, miRNAs/target genes, and the inhibitors of apoptosis are advantageous for finding the most practical approach and reducing the pathological demonstrations of lung cancer.

## Introduction

Lung cancer is the leading cause of cancer mortality, accounting for approximately 25% of all cancer deaths. Lung cancer is cancer that most often begins in the lungs but is sometimes the result of Cancer spreading from adjacent areas. Lung cancer is divided into two groups non-small cell lung cancer (NSCLC) and small cell lung cancer (SCLC) [1, 2]. Risk factors for lung cancer include two groups of genetic and environmental factors, environmental factors such as smoking, asbestos exposure, radon gas, air pollution, and age [3–6]. It is estimated that 8 to 14% of lung cancers are caused by genetic factors [7]. This genetic damage affects normal cell function, including cell proliferation, apoptosis, and DNA repair. The greater the damage, the higher the risk of cancer [8]. Lung cancer is triggered by the activation of oncogenes or the inactivation of tumor suppressor genes. Mutations in the proto-oncogene K-ras cause 10–30% of adenocarcinomas [9, 10]. Mutations and amplification of EGFR have been investigated to be common in NSCLC [11, 12]. The p53 tumor suppressor gene is affected in 60–75% of cases [13, 14]. Other genes that are often mutated or amplified include c-MET [15], NKX2-1[16], LKB1 (STK11) [17], PIK3CA [18], Bcl-2 [19, 20] and BRAF [21].

Apoptosis is a crucial pathway in regulating the cell growth rate and proliferation and the occurrence of cancers such as lung cancer which is the result of poor function or inhibition of apoptosis [22, 23]. This process is an active and energy-dependent phenomenon in which genetic mechanisms and factors play a role in controlling and executing it with special programs [24]. In the path of apoptosis, many molecules, such as microRNAs and their target genes, are involved [25–27].

MicroRNAs are non-coding ribonucleic acids and have a length of 18–25 nucleotides [28]. MicroRNAs regulate the gene expression after transcription by mRNA degradation or inhibition of their translation [29]. These molecules can act as oncogenes or tumor inhibitors in several cancers [30, 31]. MicroRNAs play a direct role in cancer through interaction with target genes and the regulation of growth, apoptosis, cell differentiation, and proliferation [32].

In the present study, we aim to study apoptosis as one of the important signaling pathways in lung cancer. Poor function or inhibition of apoptosis plays an essential role in the occurrence of lung cancer, so identifying the genes and miRNAs that control this signaling pathway can be very effective in the diagnosis, prediction, and prevention of lung cancer.

## Methods

In the present study, the genes and microRNAs involved in the apoptosis pathway of lung cancer were identified by databases and recent research studies. 100 articles were reviewed including 12 research papers and 88 review papers, which were extracted from PubMed, Web of Science, and SCOPUS databases and were published from 2000-to 2022. Additionally, the bioinformatics data were obtained using databases such as NCBI, TargetScan, UALCAN, UCSC, KEGG, miRPathDB, and Enrichr. The study is characterized in 4 sections including (1) signaling pathways of lung cancer, (2) apoptosis pathway, (3) the regulatory role of microRNAs in apoptosis, and (4) conclusion and future prospective. Following that, the IAPs and Bcl-2 pathways, which are the most important regulatory groups of apoptosis, were explained in detail. Moreover, in the section on prospects in cancer therapy, it was tried to show that the identified genes and microRNAs involved in the apoptosis pathway, can be used as Therapeutic approaches and considered appropriate biomarkers for diagnosis and prognosis of lung cancer.

## Results

### Signaling pathways of lung cancer

Control of important cellular processes, such as cell division or cell death, is the result of the function of molecules in cells that work in signaling pathways and interact with each other. The signaling pathways in lung cancer comprise:

1. RTKs pathway
2. RAS pathway
3. BRAF/MAPK pathway
4. PI3K pathway
5. LKB1/AMPK pathway,
6. TP53 pathway
7. RB1/MYC pathway
8. Wnt/β-catenin pathway
9. Epigenetic pathways
10. Oxidative stress

### Apoptosis

The process of apoptosis, as a conserved method, is under the control of genes. This process plays important role in the development and maintenance of the body by destroying old cells, unnecessary cells, and unhealthy cells, and interferes with many immune system mechanisms or diseases. This process is crucial in regulating the growth rate, cell proliferation, development, and health of the body, and the occurrence of many autoimmune diseases, cancers and viral infections is the result of poor function or inhibition of apoptosis. Therefore, the main purpose of apoptotic studies is to focus on recognizing the molecular components and regulatory mechanisms, especially the Bcl-2 family and the IAP family as the most important regulatory groups, and this information helps to apply therapeutic agents that process this pathway. They affect the treatment of neurodegenerative diseases and reproductive diseases such as cancer.

#### The mechanism of apoptosis

Apoptosis is a form of programmed cell death that occurs in multicellular organisms [33]. Caspases are a family of cysteine ​​proteases that play essential functional roles in the performance of apoptosis [34]. Mammalian caspases are functionally divided into three groups: initiator caspases (caspase 2, 8, 9, and 10), executioner caspases (caspase 3, 6, and 7), and inflammatory caspases (caspase 1, 4, 5, 11, and 12). Initiator caspases initiate the apoptosis signal while the executioner caspases conduct the mass proteolysis that leads to apoptosis [35–37]. Two main pathways of apoptosis are the Intrinsic Apoptosis Pathway and the Extrinsic Apoptosis [38].

The extrinsic pathway is initiated by the activation of death receptors such as FAS, tumor necrosis factor receptors (TNFRs), and TNF-related apoptosis-inducing ligand receptors (TRAILRs). Activation of death stimuli leads to the recruitment of death domain-containing adapter proteins such as Fas-associated protein with death domain (FADD) and TNFR1-associated death domain (TRADD). Subsequently, caspase-8, which is a downstream factor and an important mediator in the external pathway, is recruited. Finally, apoptosis begins when caspase-3 is activated by caspase-8 [33, 39–41].

**Fig. 1:**
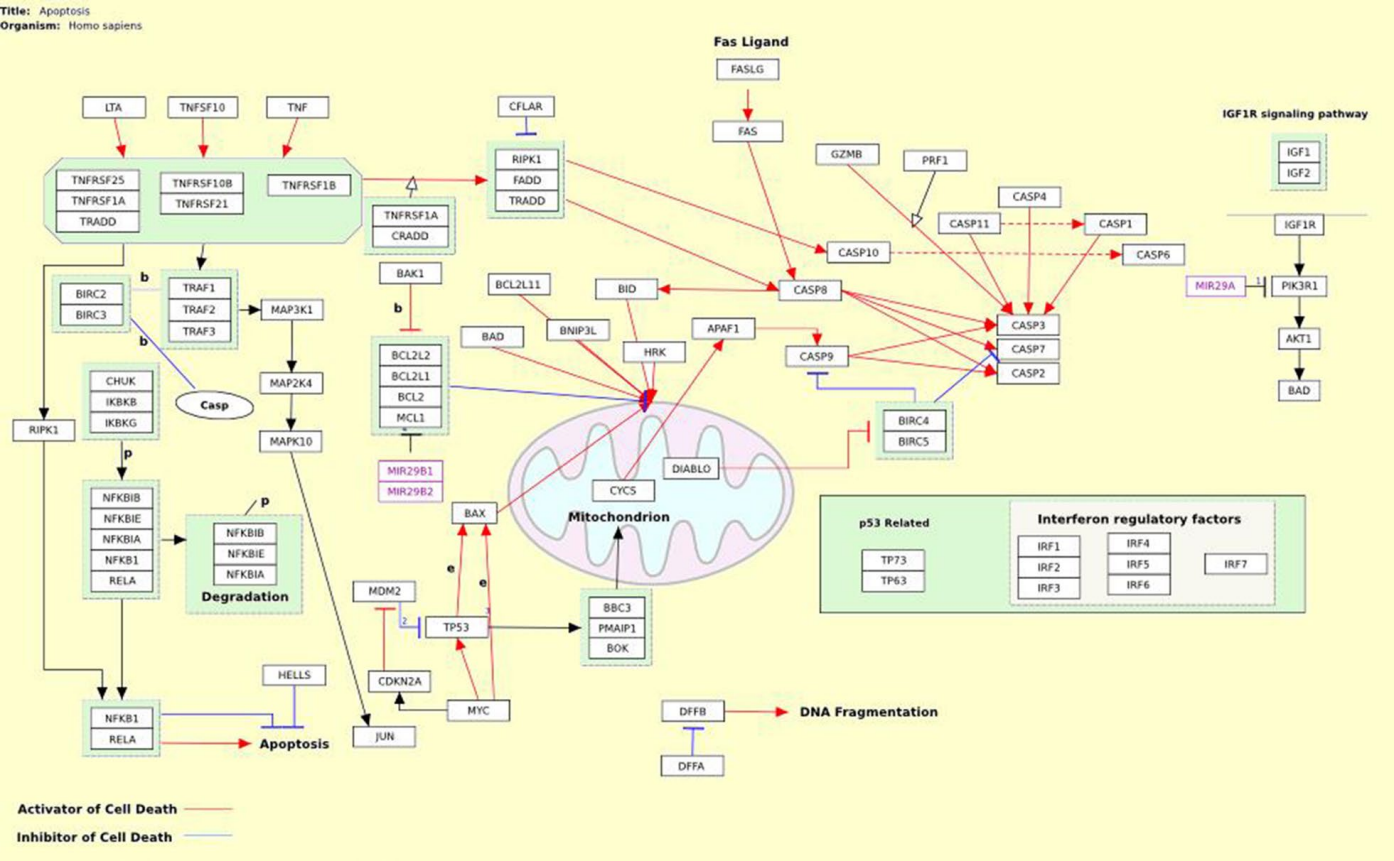
The mechanism of apoptosis signaling pathway [46]

The intrinsic Apoptosis Pathway differs from extracellular signals that cause apoptosis through the extrinsic pathway. Chemotherapy, radiotherapy, and cellular stress, including DNA damage, oxidative stress, and energy starvation, activate the Intrinsic Apoptosis Pathway [42]. In the intrinsic pathway, pro-apoptotic signaling leads to the release of cytochrome C into the cytoplasm, formation of an apoptotic complex with apoptotic protease activating factor 1 (APAF1) and the inactive form of caspase-9, activation of caspase 9, activation of caspase 3, 6 and 7 and cell apoptosis [43]. The Bcl-2 family is an important regulator of apoptosis. Bcl-2 and Bcl-XL are anti-apoptotic proteins that inhibit the secretion of cytochrome C. Whereas Bax, Bak, and Bid are pro-apoptotic proteins that release it from the mitochondria [44, 45]. Research has shown that mutations in Bcl-2 cause many cancers, especially lung cancer [45] (Fig. 1).

#### The inhibitors of apoptosis (IAPs)

**Fig. 2:**
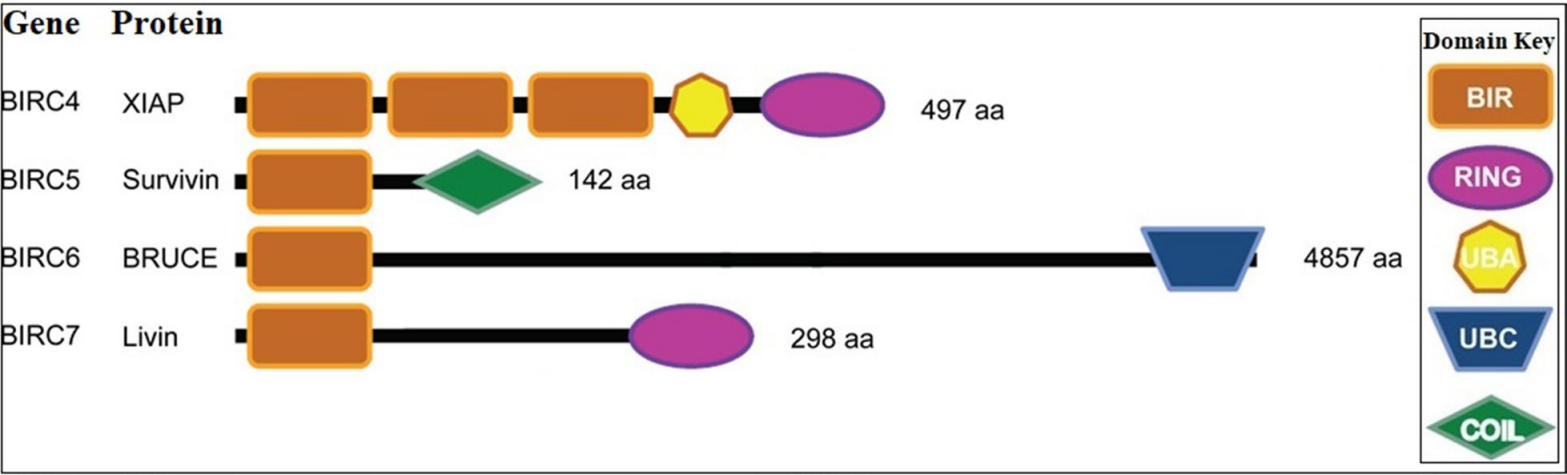
Schematic representation of human IAPs [53]. XIAP (BIRC4), Survivin (BIRC5), Livin (BIRC7), and BRUCE or Apollone ((BIRC6) are four members of the IAP family. The main cause of resistance to chemotherapy and poor prognosis of lung cancer is the overexpression of IAP proteins, which are the main culprits in escaping apoptosis

Lung cancer is the deadliest of all cancers [1, 2]. High resistance to chemotherapy and aggression has increased the need for reliable prognostic assay and effective treatments for the disease. Apoptosis is the main cellular process that plays a key role in the precise regulation of this pathway in cancer [47]. This process is regulated by several signaling pathways and three important factors affect the regulation of this process: IAP, IAP antagonists, and caspases [48, 49]. IAPs are a group of endogenous proteins that are known to control cell death and survival. These proteins play a regulatory role in apoptosis through the activation of caspases and the NF-κB signaling pathway [50, 51]. The main cause of resistance to chemotherapy and poor prognosis of lung cancer is the overexpression of IAP proteins, which are the main culprits in escaping apoptosis [52]. XIAP (BIRC4), Survivin (BIRC5), Livin (BIRC7), and BRUCE or Apollone ((BIRC6) are four members of the IAP family that play an important role in the development of chemoresistant lung cancer [51] (Fig. 2.) These data suggest that the identification of IAPs function is advantageous for finding the most practical approach and reducing the pathological demonstrations of cancers. Table 1 shows a summary of the data about IAP and IAP-based therapies which were obtained in recent clinical trials.

**Table 1:** Pre-clinical data about IAP and IAP-based therapies in clinical trials

IAP	Mechanism of inhibition	Increased sensitivity to	Refs.	Drug	Mode of action	Refs.	The regulatory miRNAs	Refs.
XIAP	Antisense	Doxorubicin Taxol Vinorelbine Etoposide	[69]	AEG35156	Antisense	[70]	miR-142	[71]
				Embelin	Small molecule targeting BIR3 domain	[72]	miR-192-5p miR-215	[73]
				Polyphenylureas/Xantags	Small molecule targeting BIR2 domain	[74]		
				Arylsulfonamides (TWX006, TWX024)		[75]		
	siRNA	Cisplatin	[64]					
Survivin	siRNA	Adriamycin Cisplatin Paclitaxol	[76] [77]	LY2181308	Antisense	[78]	miR-195	[79]
				YM155	Small molecule antagonist	[80]	miR-320	[81]
				Shepherdin	Small molecule targeting Hsp90	[82]	miR-205 miR-218	[83]
				AICAR		[84]		
				Anti-Survivin Ab	Antibody	[85]	miR-203	[86]

##### Survivin

**Fig. 3:**
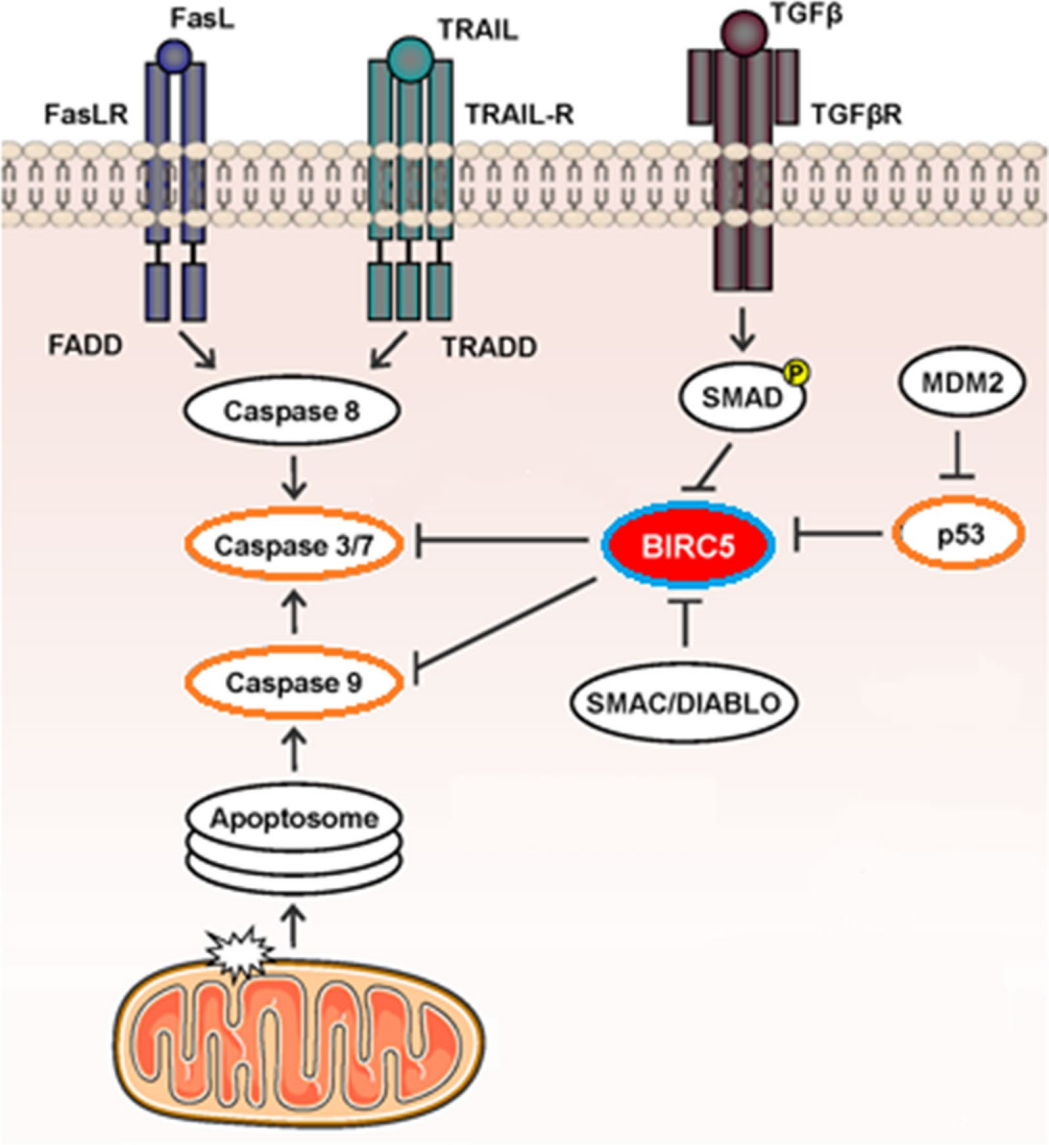
BIRC5 (survivin) acts on cytoplasm and nucleus and is involved in different cellular functions: cell survival, cell cycle progression, and apoptosis. Survivin decreases apoptosis by suppressing caspase-3, 7, and 9. Also, survivin expression is regulated by WNT/β-catenin Signaling and p53 protein; p53 represses survivin expression and the Wnt signaling pathway upregulates survivin

Survivin is a protein that is encoded by the BIRC5 gene in humans. Survivin is a member of the IAP family. This protein negatively regulates apoptosis through the inhibition of caspase3 and caspase7, which are the effector caspases in the signaling pathway of apoptosis [54, 55]. Survivin is only expressed in the G2-M phase and is highly regulated by the cell cycle [56, 57]. Moreover, the regulatory role of this protein in mitosis was shown in research [58, 59]. WNT/β-catenin Signaling and p53 protein were known to have essential roles in Survivin regulation [60–63]. Figure 3 shows the function of survivin (BIRC5) in the apoptosis pathway (http://atlasgeneticsoncology.org/).

##### XIAP

**Fig. 4:**
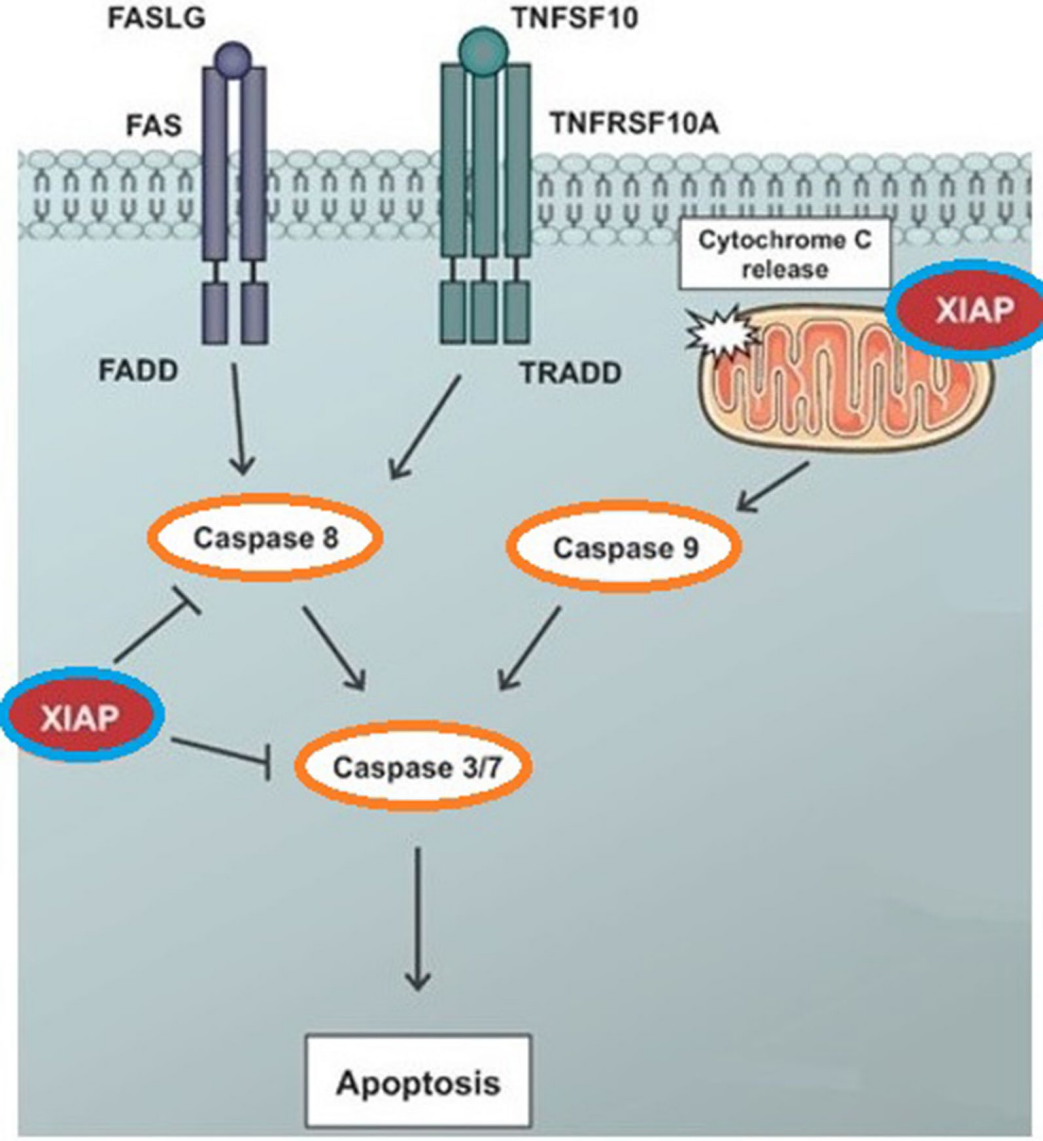
XIAP (BIRC4) is a multi-functional protein that is involved in cell death, cell cycle, cell migration, and apoptosis. The main function of XIAP is its antiapoptotic activity, which is performed by inhibiting caspase 9 and its effector's caspase 3 and caspase 7

X-linked inhibitor of apoptosis protein (XIAP) is another member of the IAP family. XIAP also known as BIRC4, is a protein that is produced by the XIAP gene. This protein is a well-known apoptosis inhibitor in cancers such as colorectal, breast, pancreatic, and lung cancer [53]. XIAP inhibits apoptosis in humans, by inhibiting the activities of caspase 9 and its effectors caspase 3 and caspase 7 [64, 65]. In addition, Smac/DIABLO and Omi/HtrA2 are the two main XIAP inhibitors. Smac/DIABLO, can enhance apoptosis by binding to XIAP and preventing it from binding to caspases. This allows normal caspase activity to proceed [66–68]. Figure 4 shows the function of XIAP (BIRC4) in the apoptosis pathway (http://atlasgeneticsoncology.org/).

### The regulatory role of microRNAs in the apoptosis

**Fig. 5:**
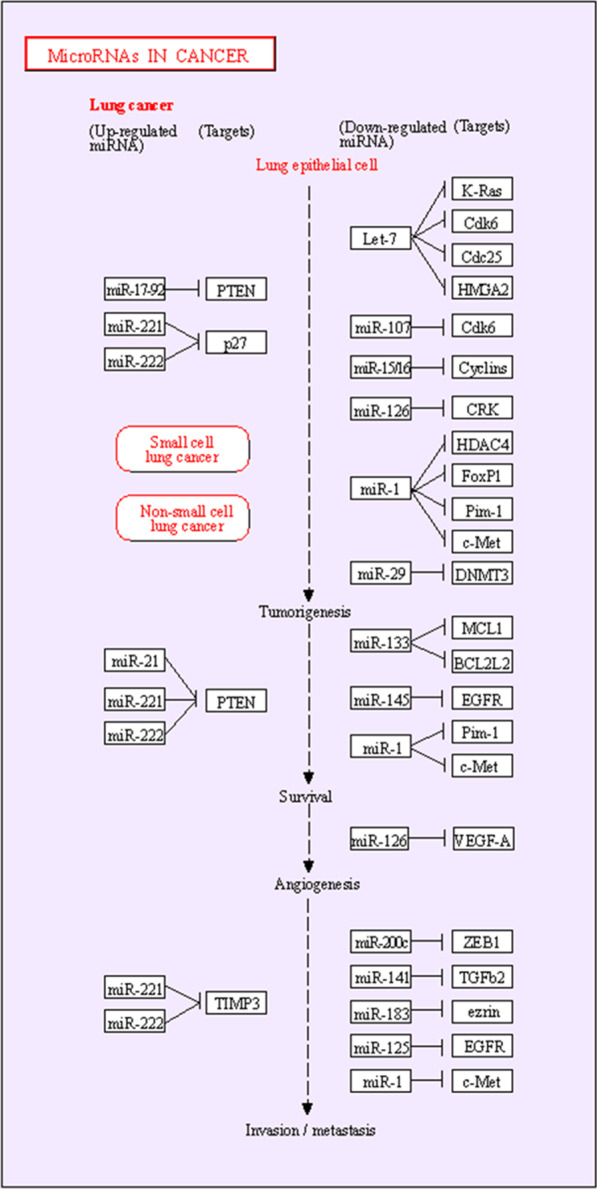
Schematic representation of the interaction between miRNAs and genes in NSCLC

The known regulatory role of microRNAs in the apoptosis pathway confirms their direct function in lung cancer. Figure 5 which was extracted from the KEGG database [87], showed the interaction of microRNAs with target genes in NSCLC. Apoptosis is involved in different stages of a living organism's biological evolution and, if left unchecked, can lead to cancer. The apoptosis signaling pathways include NF-κB, PI3K/AKT, MAPK, and P53. Many molecules, such as microRNAs are involved in the apoptosis pathway. The research and bioinformatics studies led to the identification of several microRNAs that have essential roles in the regulation of apoptosis. The involved miRNAs/Target Genes in the apoptosis signaling pathway were obtained from the miRPathDB database [88] and were shown in Table 2. In addition, the characteristics of miRNAs involved in apoptosis were extracted from miRDB [89] and the TargetScan database and were demonstrated in Table 3. MicroRNAs involved in apoptosis are classified into two categories based on their function: Proapoptotic and Antiapoptotic.

**Table 2:** The involved miRNAs/Target Genes in apoptosis signaling pathway

miRNA	P-value	Targets
hsa-miR-221-3p	0.005	APAF1,BBC3,BCL2L11,BMF,BNIP3,FOS,NAIP,TNFSF10
hsa-miR-125b-5p	0.016	BAK1,BBC3,BCL2,BCL2L2,BMF,CDKN2A,MCL1,TP53
hsa-miR-21-5p	0.004	APAF1,BCL2,CASP8, FAS,FASLG,IRAK1,MYD88,NFKB
hsa-miR-181a-5p	0.009	BAX,BCL2,BCL2L11,FOS,MCL1,XIAP
hsa-miR-146a-5p	0.011	CASP7,FADD,FAS,IRAK1,NFKB1, TRAF6
hsa-miR-139-5p	0.001	BCL2,FOS,JUN,MCL1,NFKB1
hsa-miR-181b-5p	0.014	BCL2,BCL2L11,FOS,MCL1,XIAP
hsa-let-7g-5p	0.003	BCL2L1,CASP3,CDKN2A,TNFRSF10B
hsa-miR-106b-5p	0.026	BCL2L11,CASP7,CASP8,TNFRSF10A
hsa-miR-7-5p	0.031	BAX,BCL2,FOS,XIAP
hsa-miR-133b	0.037	BCL2L1,BCL2L2,FAS,MCL1
hsa-miR-148a-3p	0.041	BAX,BCL2,BCL2L11,IKBKB
hsa-miR-582-5p	7.70e-4	CASP3,CASP9,MCL1
hsa-miR-491-5p	0.009	BCL2L1,CAPNS1,TP53
hsa-miR-146b-5p	0.012	IRAK1,NFKB1,TRAF6
hsa-miR-708-5p	0.020	BCL2,BIRC5,CASP2
hsa-miR-149-5p	0.026	BBC3,FASLG,MYD88
hsa-miR-497-5p	0.038	BCL2,BIRC5,IKBKB
hsa-let-7c-5p	0.039	BCL2L1,CASP3,TNFRSF10B
hsa-miR-224-5p	0.041	BCL2,CASP3,CASP7
hsa-miR-339-3p	0.002	MCL1,NFKB1
hsa-miR-365a-3p	0.019	BAX,BCL2
hsa-miR-630	0.027	BCL2,BCL2L2
hsa-miR-197-3p	0.034	BMF,PMAIP1
hsa-miR-33b-5p	0.037	BCL2,XIAP
hsa-miR-363-3p	0.038	BCL2L11,CASP3
hsa-miR-301a-3p	0.040	BCL2L11,MAP3K5
hsa-miR-125b-1-3p	0.044	BIK,TP53
hsa-miR-30e-5p	0.049	CASP3,TP53

**Table 3:** Characteristics of miRNAs involved in apoptosis

Signaling pathway	miRNA name	Gene symbol	Gene description	Target Score	Number of 3P-seq tags supporting UTR + 5	Total context + + score
NF-κB	MiR-146b-5p	IRAK1	Interleukin 1 Receptor Associated Kinase 1	99	54	− 0.56
	MiR-146a-5p	TRAF6	TNF receptor associated factor 6	100	91	− 1.00
	MiR-21	Bcl-2	B-cell lymphoma 2	85	1380	− 0.12
PI3K/Akt	MiR-21	PIK3CA	Phosphatidylinositol-4,5-bisphosphate 3-kinase, catalytic subunit alpha	87	112	− 0.21
	MiR-1269	PTEN	Phosphatase and tensin homolog	54	118	− 0.44
	MiR-23a			99	118	− 0.52
	MiR-181			67	118	− 0.18
	MiR-135a	Akt	AKT Serine/Threonine Kinase	70	525	− 0.21
		PIK3R2	Phosphoinositide-3-Kinase Regulatory Subunit 2	89	1484	− 0.35
	MiR-30a-5p	PIK3CD	Phosphatidylinositol-4,5-Bisphosphate 3-Kinase Catalytic Subunit Delta	93	5	− 0.09
		PIK3R2	Phosphoinositide-3-Kinase Regulatory Subunit 2	53	1484	− 0.07
MAPK	MiR-202	KRAS	KRAS Proto-Oncogene, GTPase	94	40	− 0.58
	MiR-181a	MAPK1	Mitogen-Activated Protein Kinase 1	79	630	− 0.16

#### NF-κB

MiR-21 is an Antiapoptotic miR and plays an inhibitory role in the apoptotic pathway by regulating the PI3K / Akt / NF-κB signaling pathway. Studies have shown that the miR-21 inhibitor in NSCLC cells can induce apoptosis by inhibiting the PI3K / Akt / NF-κB pathway. Induction of apoptosis by miR-21 inhibitor occurs as a result of caspase-dependent pathway regulation and increased caspase-3, 8, and 9 activities. Additionally, inactivation of Bcl-2 was observed following treatment with the miR-21 inhibitor [90].

miR-146b is another molecule that has been identified to play an inhibiting role in the apoptosis pathway. So that miR-146b inhibits IRAK4 which is an important gene in the NF-κB pathway. On the other hand, miR-146b-5p has an inhibitory effect on IL-6 and IL-8, which are NF-κB regulated chemokines, in lung cancer cells [91] .

MiR-135b is an oncogene miRNA in lung cancer that is overexpressing and enhances the invasiveness, angiogenesis, proliferation, and antiapoptosis of cancer cells. miR-135b significantly activates NF-κB. The luciferase reporter assay showed that NF-κB reporter luciferase activity in miR-135b transfected cells is higher than in control A549 cells. In addition, the upregulation of NF-κB downstream genes, such as Bcl-2, Bcl-xL, A20VEGFC, IL-1β, and IL-6 was observed as a result of miR-135b overexpression [92].

**Fig. 6:**
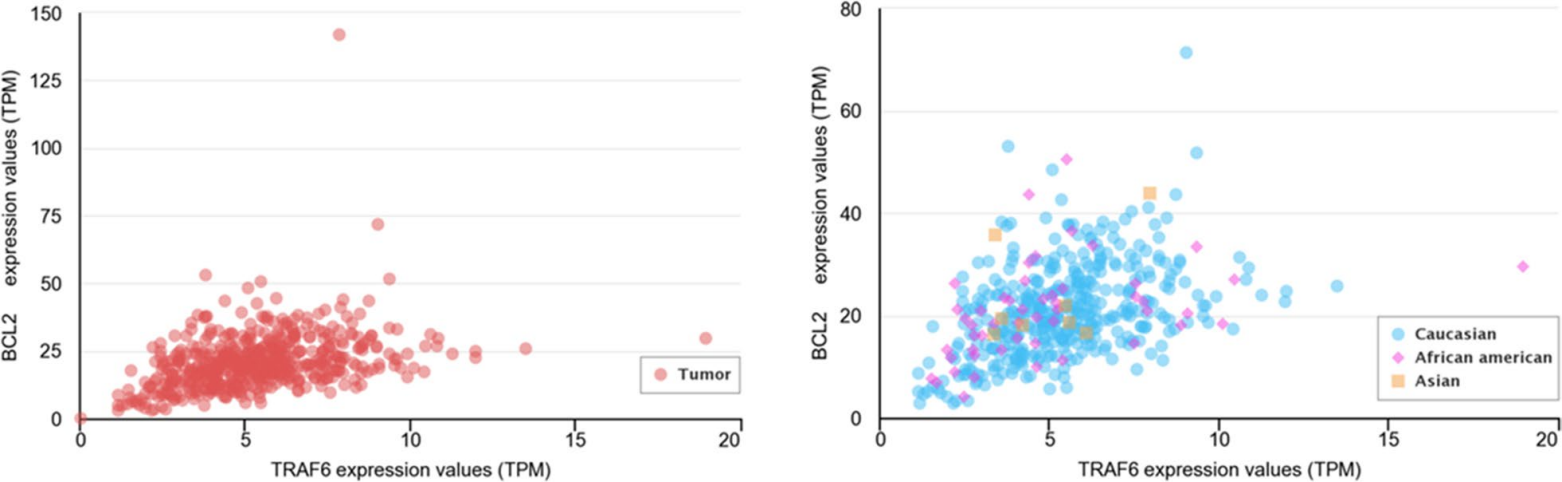
Gene expression correlation between TRAF6 and BCL-2 in lung cancer

MiR-146a-5p Inhibits apoptosis and Plays an oncogenic role in lung cancer. TRAF6 is an important gene in the NF-κB signal pathway and has a critical role in lung cancer tumorigenesis. TRAF6 is a direct target of miR-146a-5p in lung cancer cells. Therefore, miR-146a-5p binds to TRAF6 directly and suppresses this gene. MiR-146a regulates the main gene TRAF6 and continually Bcl2 via the NF-KB signaling pathway that induces apoptosis in lung cancer [93] (Fig. 6) [94]. In addition, the results of statistical analyzes in the miRDB and Target Scan databases show that miR-146a targets part 3'UTR of the TRAF6 gene in the NF-KB pathway and Bcl2 is one of the important genes involved in apoptosis.

#### PI3K/AKT

The PI3K/AKT signaling pathway is a particularly important pathway with a key role in apoptosis [95]. The PTEN gene is one of the most important genes in the apoptotic pathway. This gene acts as a suppressor tumor and increases the death of cancer cells and prevents their proliferation. Mutations in this gene are an important factor in the progression of lung cancer. Inactivation of PTEN increases the proliferation and invasion of cancer cells by activating the PI3K-AKT-NFkB signaling pathway. This gene is regulated by several microRNAs.

MiR-1269 targets PTEN directly in lung cancer cells. Using Luciferase Assay, it was shown that increasing the expression of miR-1269 in A549 cells significantly reduces the expression of PTEN compared to normal cells. In contrast, the miR-1269 inhibitor increases PTEN expression, thereby increasing the apoptosis of cancer cells [96]. In addition, Luciferase Assay in cancer stem cells (CSCs) isolated from NSCLC cells showed that PTEN is the target of miR-23a. As a result, suppression of proliferation and activation of apoptosis occurs through the activation of the PI3K-AKT pathway [97]. miR-181 regulates the PTEN / PI3K / AKT signaling pathway in A549 lung cells. Decreased expression of miR-181 suppresses the PTEN / PI3K / AKT pathway and thus activates lung cancer apoptotic cells [98]. MiR-135a promotes cell apoptosis through the IGF-1/PI3K/Akt signaling pathway in NSCLC [99]. Moreover, miR-30a-5p induces apoptosis by regulating the PI3K/AKT pathway. MiR-30a-5p inhibits the expression of PIK3R2 and PIK3CD which are two subunits of PI3K [100].

#### MAPK

The MAPK pathway (also known as the Ras-Raf-MEK-ERK pathway), plays an essential role in the apoptosis pathway. MAPKs can both activate or inhibits apoptosis depending on the cell type and the stimulus. Three subfamilies of MAPKs have been identified: extracellular signal-regulated kinases (ERKs), c-Jun N-terminal kinases (JNKs), and p38-MAPKs. The regulation of apoptosis is by JNKs and p38-MAPKs whereas, ERKs are important for cell survival [101–103]. This signaling pathway is regulated by several microRNAs.

MiR-202 inhibits the Ras / MAPK pathway by targeting the KRas gene and consequently, promotes apoptotic signaling in NSCLC A549 cells [104]. Furthermore, the upregulation of miR-181a leads to inhibition of the MAPK pathway by suppressing MAPK1 and MAP2K1 expression. This results in increased induction of apoptosis.

## Conclusion and future prospective

Apoptosis is a crucial pathway in regulating cell proliferation. So, the occurrence of many cancers such as lung cancer is the result of poor function or inhibition of apoptosis. This process is under the control of genes. In addition, the interaction of microRNAs with target genes determines their role in apoptosis and confirms the direct function of microRNAs in cancer. Some microRNAs create the oncogenic phenotype by reducing the expression of genes that suppress tumors, while others target proto-oncogenic mRNAs and turn them off, to reduce the process of becoming cancerous.

Both apoptosis and microRNAs are important in cancer. Identification of microRNAs and their target molecules has provided a clear horizon for understanding the pathways that lead to cancer. Therefore, microRNAs can be used as potential biomarkers in the diagnosis, prognosis, and treatment of cancer. With the knowledge gained from microRNAs, new therapies have been developed. Recently, the treatment of cancer cells by inserting microRNAs that are involved in apoptosis has been proposed in research. Therefore, studying oncogenes, tumor suppressor genes, miRNAs/target genes, and their associated pathways/genetic networks is advantageous for finding the most practical approach and reducing the pathological demonstrations of cancers. However, more clinical studies are needed to find the role of biomarkers in important cellular pathways such as apoptosis. In addition, understanding the diagnostic and therapeutic potential of microRNAs and target genes in cancer requires the integration of these biomarker data. According to preclinical data existing to date, using IAPs in combination with standard anti-cancer therapy yields in favorable outcome. Further extensive research validates these data on clinical grounds, and identify whether apoptosis pathway targeting miRNAs have utility as a novel class of biomarkers which can facilitate the early diagnosis, personalized treatment, and prediction of drug response for lung cancer patients.

## Data Availability

All data generated or analysed during this study are included in this published article.

## Abbreviations

NSCLC: Non-small cell lung cancer
SCLC: Small cell lung cancer
EGFR: Epidermal growth factor receptor precursor
NKX2-1: NK2 Homeobox 1
STK11: Serine/Threonine Kinase 11
PIK3CA: Phosphatidylinositol-4,5-Bisphosphate 3-Kinase Catalytic Subunit Alpha
Bcl-2: B-cell lymphoma 2
BRAF: B-Raf Proto-Oncogene, Serine/Threonine Kinase
MAPK: Mitogen-Activated Protein Kinase
FAS: Fas cell surface death receptor
TNFR: Tumor necrosis factor receptor
TRAILR: TNF-related apoptosis-inducing ligand receptors
FADD: Fas-associated protein with death domain
TRADD: TNFR1-associated death domain
APAF1: Apoptotic protease activating factor 1
BAX: BCL2 Associated X, Apoptosis Regulator
BAK: BCL2 Antagonist/Killer
Bid: BH3 Interacting Domain Death Agonist
IAP: Inhibitors of apoptosis
BIRC: Baculoviral IAP repeat-containing protein
NF-κB: Nuclear factor kappa-light-chain-enhancer of activated B cells
TRAF6: TNF receptor associated factor 6
PTEN: Phosphatase and tensin homolog
PI3K: Phosphatidylinositol-4,5-Bisphosphate 3-Kinase
ERK: Extracellular signal-regulated kinas
JNK: C-Jun N-terminal kinas
FASLG: Fas Ligand (TNF Superfamily, Member 6)
TNFSF10: TNF Superfamily Member 10
